# Studying User Income through Language, Behaviour and Affect in Social Media

**DOI:** 10.1371/journal.pone.0138717

**Published:** 2015-09-22

**Authors:** Daniel Preoţiuc-Pietro, Svitlana Volkova, Vasileios Lampos, Yoram Bachrach, Nikolaos Aletras

**Affiliations:** 1 Positive Psychology Center, University of Pennsylvania, Philadelphia, PA, United States of America; 2 Center for Language and Speech Processing, Johns Hopkins University, Baltimore, MD, United States of America; 3 Computer Science Department, University College London, London, United Kingdom; 4 Microsoft Research, Cambridge, United Kingdom; IFIMAR, UNMdP-CONICET, ARGENTINA

## Abstract

Automatically inferring user demographics from social media posts is useful for both social science research and a range of downstream applications in marketing and politics. We present the first extensive study where user behaviour on Twitter is used to build a predictive model of income. We apply non-linear methods for regression, i.e. Gaussian Processes, achieving strong correlation between predicted and actual user income. This allows us to shed light on the factors that characterise income on Twitter and analyse their interplay with user emotions and sentiment, perceived psycho-demographics and language use expressed through the topics of their posts. Our analysis uncovers correlations between different feature categories and income, some of which reflect common belief e.g. higher perceived education and intelligence indicates higher earnings, known differences e.g. gender and age differences, however, others show novel findings e.g. higher income users express more fear and anger, whereas lower income users express more of the time emotion and opinions.

## Introduction

The vast amount of publicly available user-generated content on social media enables the study of complex problems for which sufficient data was not available before in various domains, such as health, politics or economics [1–3]. Automatic analysis of that content can unveil interesting patterns of language [4] and infer characteristics of users. Examples range from location [5], gender [6], age [7, 8], personality [9] or political preference [10, 11]. Usually, inferring user characteristics is framed as a predictive task validated on held-out data. This is solved by established regression or classification methods or more sophisticated latent variable models tailored to the task [10, 12]. Conversely, some studies also provide an analysis of the predictive linguistic variables with the purpose to unveil sociological insight [13, 14]. Automatically inferred user characteristics can enable large-scale social science studies or assist applications such as targeted advertising, polling across different demographics or sentiment analysis [15].

This is the first study on automatically inferring the income of social media users. An income predictor is a useful tool for exploring the important effect of socio-economic status in subsequent social science studies using Twitter data, e.g. in public health applications. Moreover, such information can be used in mainstream commercial applications, e.g. in personalised advertising. We hypothesise that income is revealed through a variety of factors, starting from the actual text posted by a user [16], but also via other information, such as the number of friendships, demographics (e.g. gender and age), personality [17], education level or expressed emotions. Previous related studies on income originated in socio-economic research. Income of people has been predicted using demographic features such as the congressional district in which the respondent lived, educational categories, sex, age, age squared, race categories, marital status categories, and height [18]. Another study showed that psychological traits related to extraversion (e.g. larger social networks) and conscientiousness (e.g. orderliness) have positive correlation with income, while neurotic traits (e.g. anger, anxiety) are anti-correlated [19].

We frame the income prediction task as regression using linear as well as non-linear learning algorithms. For training and testing, we use a large dataset of Twitter users annotated with their income, using fine-grained user occupation as a proxy. For prediction we use a broad spectrum of features, ranging from simple user profile features (e.g. number of followers) to inferred psycho-demographics, emotions and word topics. Experimental results show that income is highly predictable given the content generated by a user, with the best non-linear models reaching up to .633 Pearson correlation.

The other goal of this work is to give insights into the features that correlated with income on Twitter. Therefore, we conduct a qualitative analysis by examining the output and the parametrisation of our regression models. The most important features are identified by using the Bayesian non-parametric framework of Gaussian Processes (GPs), which supports non-linear modelling as well as interpretability through the use of Automatic Relevance Determination (ARD) [20]. Taking advantage of this property, we expose the relationship between income and attributes such as language use, platform behaviour or affect.

Our approach replicates broadly accepted norms or statistically supported trends such as income being correlated with perceived education, intelligence and age as well as the difference in pay between males and females. In addition, a number of more intriguing patterns are uncovered. Users perceived as religiously unaffiliated and less anxious appear to have higher earnings. These users have more followers and get retweeted more, albeit following similar number of persons, tweeting less and with fewer URLs. Automatic analysis of language use uncovered that higher income users express more anger and fear while posting less subjective content—both positive and negative. Finally, the topics were identified as the best predictors, with higher income users posting more about politics, non-governmental organisations (NGOs) and corporate topics, while lower income users adopting more swear words.

## Materials and Methods

### Data

We create a large dataset consisting of Twitter users mapped to their income, together with their platform statistics and historical tweet content. This dataset is based on mapping a Twitter user to a job title and—using this as a proxy—to the mean income for that specific occupation.

We use a standardised job classification taxonomy for mapping Twitter users. The Standard Occupational Classification (SOC) [21, 22] is a UK government system developed by the Office of National Statistics (ONS) for listing and grouping occupations. Jobs are organised hierarchically based on skill requirements and content.

The SOC taxonomy includes nine 1-digit groups coded with a digit from 1 to 9. Each 1-digit group is divided into 2-digit groups, where the first digit indicates its 1-digit group. Each 2-digit group is further divided into 3-digit groups and finally, 3-digit groups are divided into 4-digit groups. The 4-digit groups contain specific jobs together with their respective titles. Table 1 shows a part of the SOC taxonomy. In total, there are 9 1-digit groups, 25 2-digit groups, 90 3-digit groups and 369 4-digit groups. Although other occupational taxonomies exist, we use SOC because it has been updated recently (2010), is the outcome of years of research [22], contains newly introduced jobs, has a balanced hierarchy and offers a wide variety of job titles that were crucial in our dataset creation. A recent study has proven the effectiveness of building large corpora of users and their SOC occupation from social media finding many similarities to real world population distribution across jobs [23].

**Table 1:** Subset of the SOC classification hierarchy.

**Group 112**: Production Managers and Directors (50,952 GBP/year)
•Job titles: engineering manager, managing director, production manager, construction manager, quarry manager, operations manager
**Group 241**: Conservation and Environment Professionals (53,679 GBP/year)
•Job titles: conservation officer, ecologist, energy conservation officer, heritage manager, marine conservationist, energy manager, environmental consultant, environmental engineer, environmental protection officer, environmental scientist, landfill engineer
**Group 312**: Draughtspersons and Related Architectural Technicians (29,167 GBP/year)
•Job titles: architectural assistant, architectural, technician, construction planner, planning enforcement officer, cartographer, draughtsman, CAD operator
**Group 411**: Administrative Occupations: Government and Related Organisations (20,373 GBP/year)
•Job titles: administrative assistant, civil servant, government clerk, revenue officer, benefits assistant, trade union official, research association secretary
**Group 541**: Textiles and Garments Trades (18,986 GBP/year)
•Job titles: knitter, weaver, carpet weaver, curtain maker, upholsterer, curtain fitter, cobbler, leather worker, shoe machinist, shoe repairer, hosiery cutter, dressmaker, fabric cutter, tailor, tailoress, clothing manufacturer, embroiderer, hand sewer, sail maker, upholstery cutter
**Group 622**: Hairdressers and Related Services (10,793 GBP/year)
•Job titles: barber, colourist, hair stylist, hairdresser, beautician, beauty therapist, nail technician, tattooist
**Group 713**: Sales Supervisors (18,383 GBP/year)
•Job titles: sales supervisor, section manager, shop supervisor, retail supervisor, retail team leader
**Group 813**: Assemblers and Routine Operatives (22,491 GBP/year)
•Job titles: assembler, line operator, solderer, quality assurance inspector, quality auditor, quality controller, quality inspector, test engineer, weightbridge operator, type technician
**Group 913**: Elementary Process Plant Occupations (17,902 GBP/year)
•Job titles: factory cleaner, hygene operator, industrial cleaner, paint filler, packaging operator, material handler, packer

**Fig 1:**
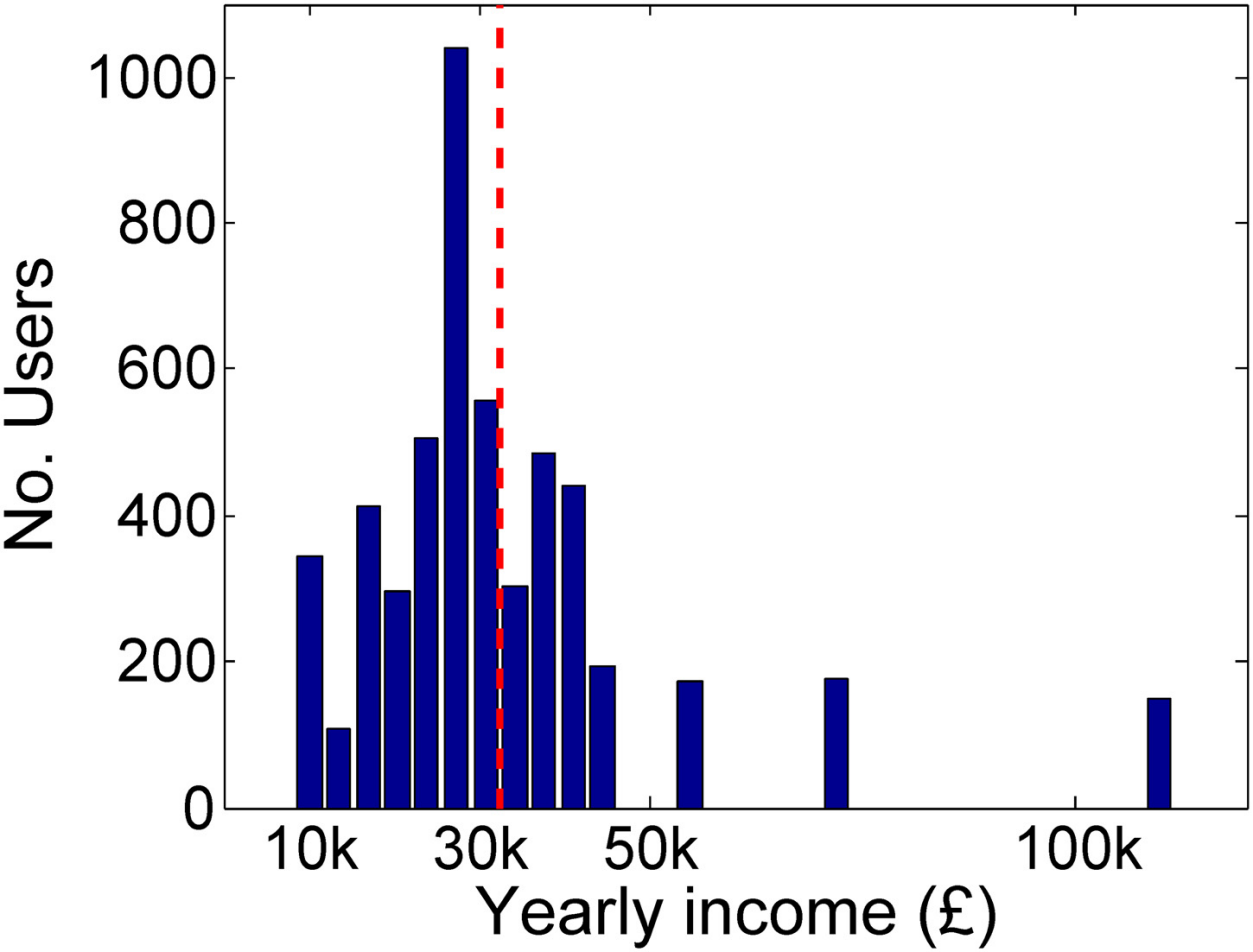
The distribution of yearly income for the users in our dataset. The red dotted line represents the mean.

We use the job titles provided by the extended description of each 4-digit SOC groups to query the Twitter Search API and retrieve a maximum of 200 accounts which best matched each job title. In order to clean our dataset of inevitable errors caused by keyword matching (e.g. ‘coal miner’s daughter’ is retrieved using the ‘coal miner’ keywords) two of the authors performed a manual filtering of all retrieved profile descriptions. We removed all profiles where either of the annotators considered that the profiles were not indicative of the job title (e.g. ‘spare time guitarist’), contained multiple possible jobs (e.g. ‘marketer, social media analyst’) or represented an institutional account (e.g. ‘limo driver company’). In total, around 50% of the accounts were removed by manual inspection performed by the authors. Finally, we removed all 3-digit categories that contained less than 50 user accounts after filtering. This resulted in a total number of 5,191 users from 55 3-digit groups, spread across all nine 1-digit SOC groups. We use the Annual Survey of Hours and Earnings [24] released by the Office for National Statistics of the UK to map each user to the mean yearly income for 2013 in British Pounds (GBP) for its 3-digit job class. The distribution of user income in our dataset is presented in Fig 1.

For the users in the dataset, we have collected all of their tweets, going back as far as the latest 3,200, and their platform statistics. The final dataset consists of 10,796,836 tweets collected around 5 August 2014 and is freely available [25]. The data was preprocessed, i.e. tokenisation and language identification, using the Trendminer pipeline [26].

### User Features

The first group of features used in our experiments are user level properties either extracted directly from a user’s profile or via established classifiers which can infer latent user characteristics from text information. They are presented in the following paragraphs.

#### Profile Features (Profile)

Profile features comprise of statistics computed based on the user’s profile information. Table 2a presents the eight features in this category.

**Table 2:** Description of the user level features.

**(a)** User profile features (Profile)	
*u*_1_	number of followers
*u*_2_	number of friends
*u*_3_	number of times listed
*u*_4_	follower/friend ratio
*u*_5_	no. of favourites the account made
*u*_6_	avg. number of tweets/day
*u*_7_	total number of tweets
*u*_8_	proportion of tweets in English
**(b)** User psycho-demographic features (Demo)	
*d*_1_	gender (male, female)
*d*_2_	age (18–70)
*d*_3_	political (independent, conservative, liberal, unaffiliated)
*d*_4_	intelligence (> average, average, ≤ average, ≫ average, ≪ average)
*d*_5_	relationship (married, in a relationship, single, other)
*d*_6_	ethnicity (Asian, African American, Indian, Hispanic, Other, Caucasian)
*d*_7_	education (bachelor, graduate, high school)
*d*_8_	religion (Christian, Jewish, Muslim, Hindu, unaffiliated, other)
*d*_9_	children (yes, no)
*d*_10_	income (below average, above average, very high)
*d*_11_	life satisfaction (satisfied, dissatisfied, very satisfied, very dissatisfied, neither)
*d*_12_	optimism (optimist, pessimist, extreme optimist, extreme pessimist, neither)
*d*_13_	narcissism (agree strongly, agree, disagree, disagree strongly, neither)
*d*_14_	excited (agree strongly, agree, disagree, disagree strongly, neither)
*d*_15_	anxious (agree strongly, agree, disagree, disagree strongly, neither)
**(c)** User emotion features (Emo)	
*e*_1_	proportion of tweets with positive sentiment
*e*_2_	proportion of tweets with neutral sentiment
*e*_3_	proportion of tweets with negative sentiment
*e*_4_	proportion of joy tweets
*e*_5_	proportion of sadness tweets
*e*_6_	proportion of disgust tweets
*e*_7_	proportion of anger tweets
*e*_8_	proportion of surprise tweets
*e*_9_	proportion of fear tweets
**(d)** Shallow textual features (Shallow)	
*s*_1_	proportion of non-duplicate tweets
*s*_2_	proportion of retweeted tweets
*s*_3_	average no. of retweets/tweet
*s*_4_	proportion of retweets done
*s*_5_	proportion of hashtags
*s*_6_	proportion of tweets with hashtags
*s*_7_	proportion of tweets with @-mentions
*s*_8_	proportion of @-replies
*s*_9_	no. of unique @-mentions in tweets
*s*_10_	proportion of tweets with links

#### Inferred Perceived Psycho-Demographic Features (Demo)

User psycho-demographic features are automatically inferred based on user’s published text using the methods developed in [27]. These represent logistic regression models trained on binary features containing unique word types (also known as unigrams) extracted from tweets. The models have been trained on 5,000 Twitter profiles annotated with perceived user properties obtained through crowdsourcing. In total, for each user we predict 15 psycho-demographic features described in Table 2b.

Crowdsourcing perceived attribute annotations—collecting the subjective impressions workers get when reading the content of user profiles—is not trivial [28, 29]. For building the models used in this paper, labeled data was obtained using a group of trained and screened workers who reside in the US, have over 98% reputation score and have been involved in similar annotation tasks on Amazon Mechanical Turk before. In addition, a variety of quality control questions with known ground truth were embedded and the workers had to answer all of them correctly for their work to be accepted. To reduce annotator bias (e.g. users generating more emotional tweets perceiv0ed to be females), the workers were **given access to complete Twitter profiles including user bio, tweets, photos and videos**. The large scale of the annotation effort resulted in a high monetary cost for annotating 5,000 profiles on multiple traits. Thus, to minimise the annotation cost a single annotation was obtained per target user profile. To measure the degree of agreement between raters, we collected redundant annotations for a 2% random subsample of user profiles. The inter-annotator agreement measured using Cohen’s kappa ranges between fair (0.3 < *κ* < 0.7 for the majority of subjective attributes) and high (*κ* > 0.7 for gender and ethnicity attributes).

To ensure the quality of subjective annotations we tested the models trained on crowdsourced labels on publicly available data from [11, 30] for classifying gender and political orientation. Our models yield significantly higher performance (up to 10% accuracy gain) which highlight the quality of crowdsourced annotations. In addition, recent research looked more deeply into classifying political orientation, analyzed data annotation and sampling biases in social media and showed how classification accuracy depends on the amount of data available for prediction [11, 31]. They showed that political orientation classifiers achieve accuracy between 63%—91%. For previously unexplored attributes (e.g. education, relationships status, optimism), prediction quality measured using 10-fold cross validation as ROC AUC (the probability of correctly classifying two randomly selected profiles of each of the two most frequent classes) ranges between 0.63 (religion)—0.93 (ethnicity) [27].

Note that in our regression experiments we are using the probability distribution of a user over all possible classes rather than the actual class label. This allows to capture more information and limits class imbalance issues in our training data. Age and gender are predicted using text-based models described in [32]. These models were trained on data from over 70,000 Facebook users and reports an accuracy of 91.9% for gender (88.9% on Twitter data) and Pearson correlation *r* = 0.84 for age prediction.

#### Emotions (Emo)

In addition, Table 2c outlines nine emotion features: the six Ekman’s emotions [33] and three sentiment (valence) scores, all automatically inferred from user tweets. To obtain these, we use predictive models based on binary unigram features and a set of affect features from [27]. These text-based models predict one of six emotions—joy, sadness, fear, disgust, surprise and anger—and one of three valence features—positive, negative, neutral—for each tweet. We aggregate all emotions and sentiment per user and calculate the proportion of every emotion and sentiment per user.

### Textual Features

We derive textual features from the Twitter posts of each user. These are either shallow features or deeper semantic topics.

#### Shallow Features (Shallow)

The first set of textual features are shallow statistics on user’s texts presented in Table 2d.

#### Word Clusters (Topics)

In text regression tasks, textual features are usually represented by a list of unigrams. Each feature represents the number of times that a word found in the tweets of a particular user. Although useful, this study focuses on using a more abstract representation of textual features with the goal of interpretability.

To build the clusters we use a separate Twitter dataset consisting of the Twitter Gardenhose stream (a 10% sample of the entire Twitter stream) from 2 January to 28 February 2011. We first create a list of the most frequent unigrams (71,555) and then we obtain their vector representations. Neural language models learn low dimensional vectors for words through a hidden layer of a neural network. Dense word vectors, i.e. embeddings, are computed using a state-of-the-art model, Word2Vec [34, 35]. We use the skip-gram model with negative sampling [35] to learn the embeddings from the Twitter reference corpus using Gensim [36]. The dimensionality of the embeddings is set to 50. A detailed description of neural language models is presented in [34, 35].

Then, we group together words into clusters or *topics*, i.e. words that are semantically or syntactically similar, using their embeddings. We derive a word by word matrix where each row and column represent a word and each cell represents the cosine similarity between their embeddings. We finally apply spectral clustering on that matrix to obtain the 200 distinct word clusters. The clusters are interpreted by a list of the top representative words—those with the highest average relatedness to the rest of the words in the cluster. For each user, we aggregate all tokens in his tweets and represent the user as a distribution over the clusters, normalised by the number of tweets. We have selected this configuration after trying several other clustering methods (i.e. LDA [37], GloVe embeddings [38], NPMI clustering [13]) and number of clusters (ranging from 30 to 2000).

### Predictive Models

We frame our income prediction task as regression using a combination of user level and textual features. We use state-of-the-art linear and non-linear methods. The linear method is the logistic regression (**LR**) [39] with Elastic Net regularisation [40]. The first non-linear method is Support Vector regression [41] (**SVM**) with a Radial Basis Function (RBF) kernel [42], as implemented in the Scikit Learn Toolkit [43]. Although a standard non-linear method used in regression, SVMs do not inform which features are the most important in our predictive task. For this reason, we use Gaussian Processes (GP) [20] for regression. GPs formulate a Bayesian non-parametric statistical framework which defines a prior on functions. The properties of the functions are given by a kernel which models the covariance in the response values as a function of its inputs. In order to enable feature interpretability, we use the Squared Exponential (a.k.a. RBF) covariance function with Automatic Relevance Determination (ARD) [44] to learn a separate kernel lengthscale for each feature. Intuitively, the lengthscale parameter controls the variation along that dimension, i.e. a low value makes the output very sensitive to input data, thus making that input more useful for the prediction. Given that our dataset is very large and number of features high, for GP inference we use the fully independent training conditional (FITC) approximation [45] with 500 random inducing points. Finally, we combine the outcomes of the models learned using all feature sets in a linearly weighted ensemble.

## Results and Discussion

### Income Prediction

We first measure the predictive power of our features by performing regression on the user income. Performance is measured using 10 fold cross-validation: in each round, 80% of the data is used to train the model, 10% is used to tune model parameters using grid search (for LR and SVM) and a different 10% is held out for testing. The final results are computed over the aggregate set of results of all 10 folds. Results using all three regression methods and all types of features are presented in Table 3. Performance is measured using two standard metrics: Pearson’s correlation coefficient *r* and Mean Absolute Error (MAE) between inferred and target values.

**Table 3:** Prediction of income with our groups of features. Pearson correlation (left columns) and Mean Average Error (right columns) between income and our models on 10 fold cross-validation using three different regression methods: Linear regression (LR), Support Vector Machines with RBF kernel (SVM) and Gaussian Processes (GP) and sets of features described in the User Features section.

**Feature set**	**No. Features**	**LR**		**SVM**		**GP**	
Profile	8	.205	*£*11460	.331	*£*11033	.372	*£*11291
Demo	15	.278	*£*11126	.257	*£*10418	.364	*£*10110
Emo	9	.271	*£*11093	.358	*£*10768	.371	*£*10980
Shallow	10	.200	*£*11183	.261	*£*11494	.355	*£*11456
Topics	200	.498	*£*10430	.606	*£*9835	.608	*£*9621
All features (Linear ensemble)	5	.506	*£*10342	.614	*£*9652	.633	*£*9535

Best results are obtained using a combination of all features, reaching a correlation of .633 and Mean Average Error of *£*9535 with user income. This shows that our models predict income with high accuracy. In general, the non-linear methods outperform the linear methods with wide margins, showing the importance of modelling non-linear relationships in our data. Out of all sets of features, the best results are obtained using the word clusters, with the other four sets of features having each similar predictive power. The strong predictive performance allows us to study more in depth the impact of all features while automatically inferring user income.

### Psycho-Demographics

**Fig 2:**
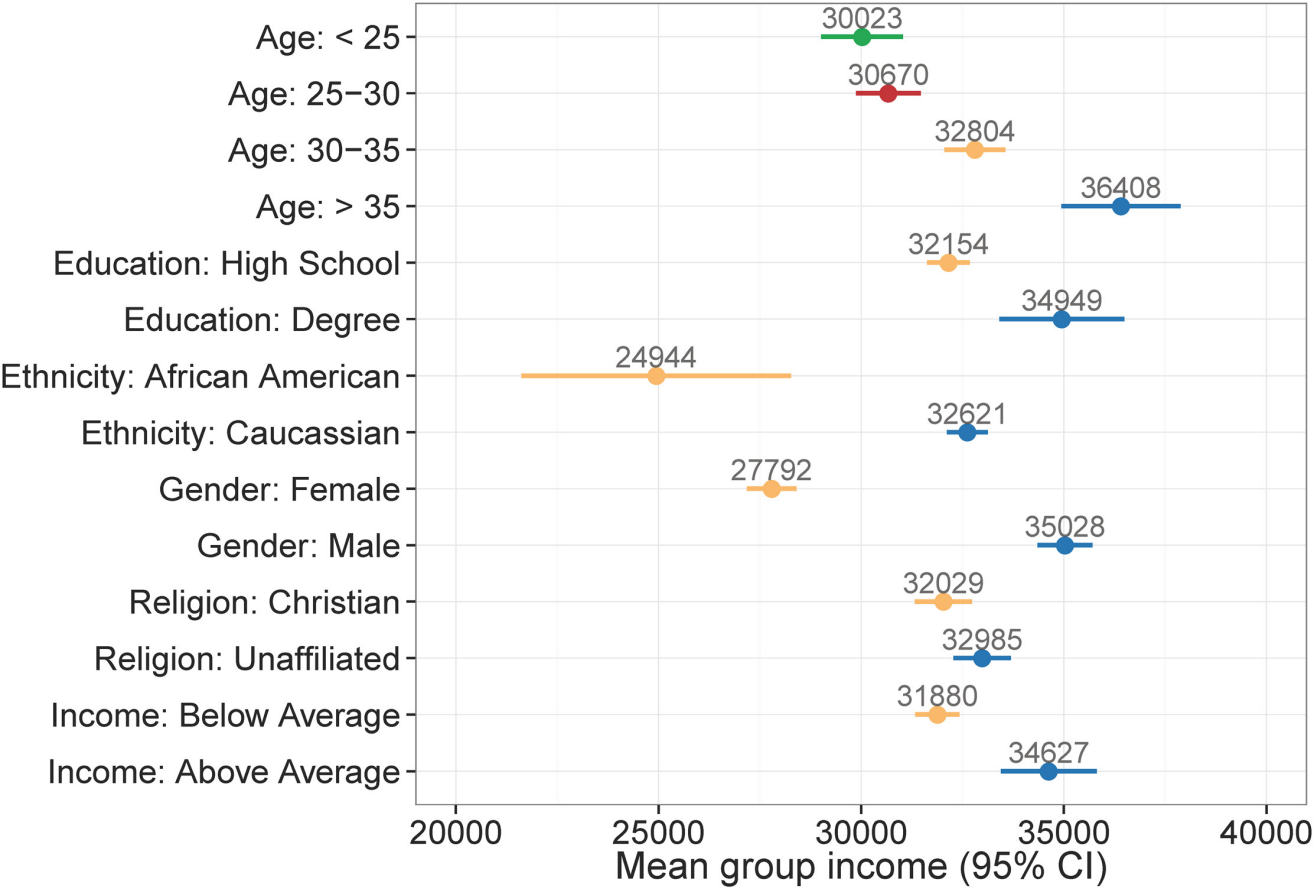
Mean income with confidence intervals for psycho-demographic groups. All group mean differences are statistically significant (Mann-Whitney test, *p* < .001).

We begin our analysis by examining the perceived psycho-demographic features. The psycho-demographic features are categorical—unlike all others—and allow us to compare variations within groups. To relate income with perceived psycho-demographic attributes we compare the average income within the groups of users with contrastive psycho-demographic attributes. Fig 2 shows the average income for groups with mean differences statistically significant at *p* < .001 (Mann-Whitney test [46]).

The following findings confirm known relationships and establish the validity of our automatic approach and dataset:

- For gender, there is a well-known payment gap [47], with average males earning significantly more than females. For example, the gender pay ratio in the US is .816 [48] and in the UK is 0.906 [49]. In our dataset, the mean ratio between females and males is .793;
- Age plays an important role in income, with older age groups earning on average significantly more than younger ones, reaching a plateau after the age of 35 [48]. Higher age leads on average more work experience and education, which is translates to a higher income;
- There is a very strong racial difference in income level [50], with the mean ratio between African Americans and Caucassians being for example .784 in the US [48]. In our dataset, users perceived as African Americans earn much less (*£*24,944) on average than Caucasians (*£*32,621) with a mean ratio of .764. This large gap can be partly explained with the perceived nature of our race predictions, with African American language markers associated indirectly with lower social status and income.
- Higher perceived education plays a significant role in having higher income;
- Differences in real income between predicted perceived income groups are significant. We highlight that other groups (e.g. high income or graduate studies) have few users assigned and therefore it is hard to estimate a reliable group mean;
- Predicted perceived intelligence should be correlated with actual income. However, the vast majority of people are predicted to be part of the average intelligence class. Annotating intelligence from text is a hard task and our classifier was trained on labels which had a very low Cohen’s *κ* = .07 [51]. However, predicting actual income using only perceived intelligence probabilities still leads to correlations (.135).

In addition, we unveil the following relationships on Twitter:

- Users perceived as being Christian earn significantly less on average than people who chose not to signal their religious belief. This is different to surveys in the US [52] which show that income levels are very similar between Christians and non-affiliated. This finding is caused by users who are perceived of being Christian from their posts earn significantly less than users who do not disclose their religious beliefs;
- Perceived anxiety and optimism differences in means were observed at higher p-values (.05). Perceived optimists had a lower mean income (*£*32,267) than users neither optimist or pessimist (*£*32,678), while less anxious users had higher mean income (*£*32,608) than users perceived neither anxious nor calm (*£*31,337). Higher income social media users do not signal anxiousness, although wealth has been found to have a positive correlation with stress [53];
- For narcissism and excitability groups incomes are not significantly different, but results show trends by which narcissistic and excitable persons have lower income than those who do not express these traits (*p* < .07);
- Other psycho-demographic features such as political orientation or life satisfaction are not significantly related to income. The relationship between life satisfaction and income is widely debated [54].

Subsequently, we analyse the distribution of psycho-demographic traits in a balanced sample of 1,000 users with the highest (*£*53,679—*£*111,413) and lowest (*£*8,395—*£*16,035) income. For each trait we estimate and compare the probability of every attribute given the income classes e.g. *p*(Female ∣ *i* = High) vs. *p*(Female ∣ *i* = Low). We test whether these attribute values are statistically significant using a Mann-Whitney test (*p* < .001). The significant differences for higher compared to lower income are (↑ —higher, ↓ —lower—income group): users > 35 y.o. 18%↑, 30—35 y.o. 14%↑, 25—30 y.o. 12%↓, < 25 y.o. 20%↓; female 31%↓; Christians 5%↓; Caucasians 4%↑; users with a degree 7%↑ and users with above average intelligence 2%↑; narcissists 3%↓. We did not find any significant differences for other attributes such as political preference and relationship status.

### Profile

**Fig 3:**
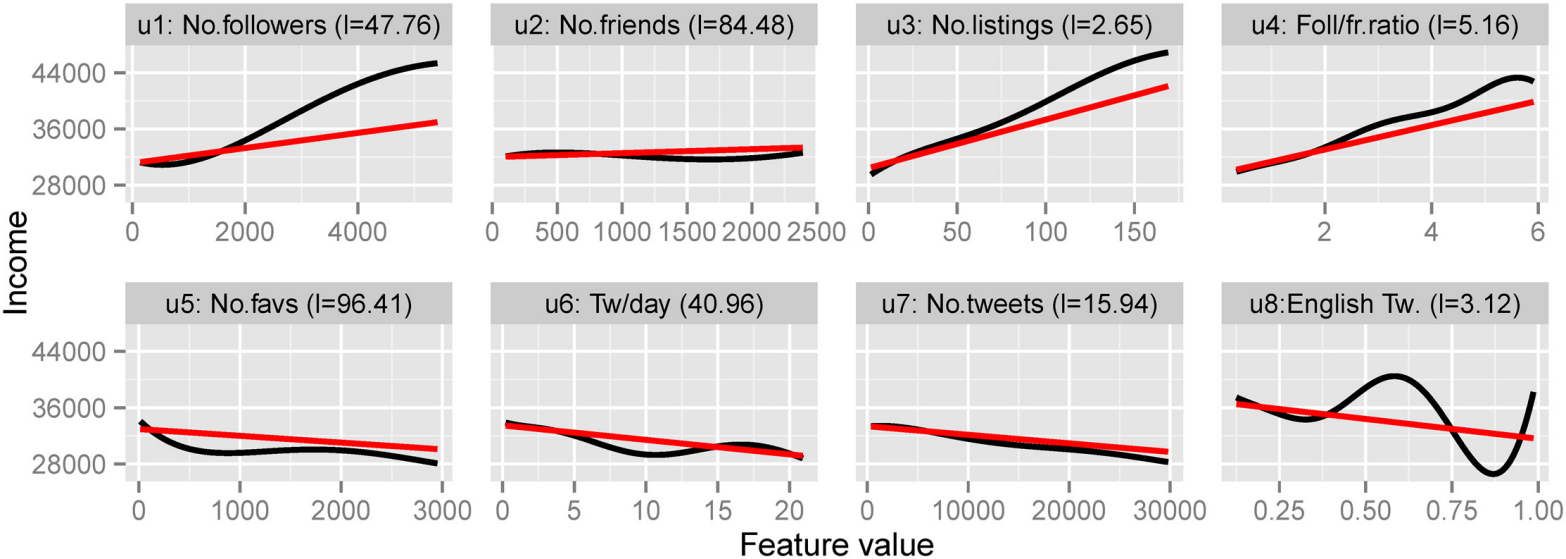
Linear and non-linear (GP) fit for Profile features. Variation of income as a function of user profile features. Linear fit in red, non-linear Gaussian Process fit in black. Brackets show the GP lengthscales—the lower the value, the more important the feature is for prediction.

We interpret the non-linear relationship between the rest of the features and income using Gaussian Processes. The GP’s lengthscale parameters are inversely proportional to feature relevance. We can use the lengthscales to rank feature importance for the prediction task. Furthermore, we can use the lengthscale for each feature to fit a GP to the feature values and plot the non-linear function. Because the lengthscales are uncorrelated—unlike linear regression—we use for interpretation a model which combines all features exhibiting a non-linear relationship (Topics, Profile, Shallow, Emo). This allows us to rank the importance within each feature type as well as between the types. In Fig 3 we plot the relationship between all profile features and user income together with the lengthscale for each feature.

We observe the following trends:

- Higher income users have significantly more followers (*u*_1_), albeit the number of friends (*u*_2_) is not dependent on income. The most predictive feature is the number of times a user is listed (*u*_3_). Twitter listing is a way to organise users into lists for easier following and is perceived as a higher form of endorsement than following that user. Higher income users are listed more than lower income users;
- The rate of Twitter posting (*u*_6_, *u*_7_) increases as the income gets lower in a near linear relationship. This could be caused by the fact that lower income users use Twitter more for social interaction (which leads to higher volumes of posts).

### Emotions

**Fig 4:**
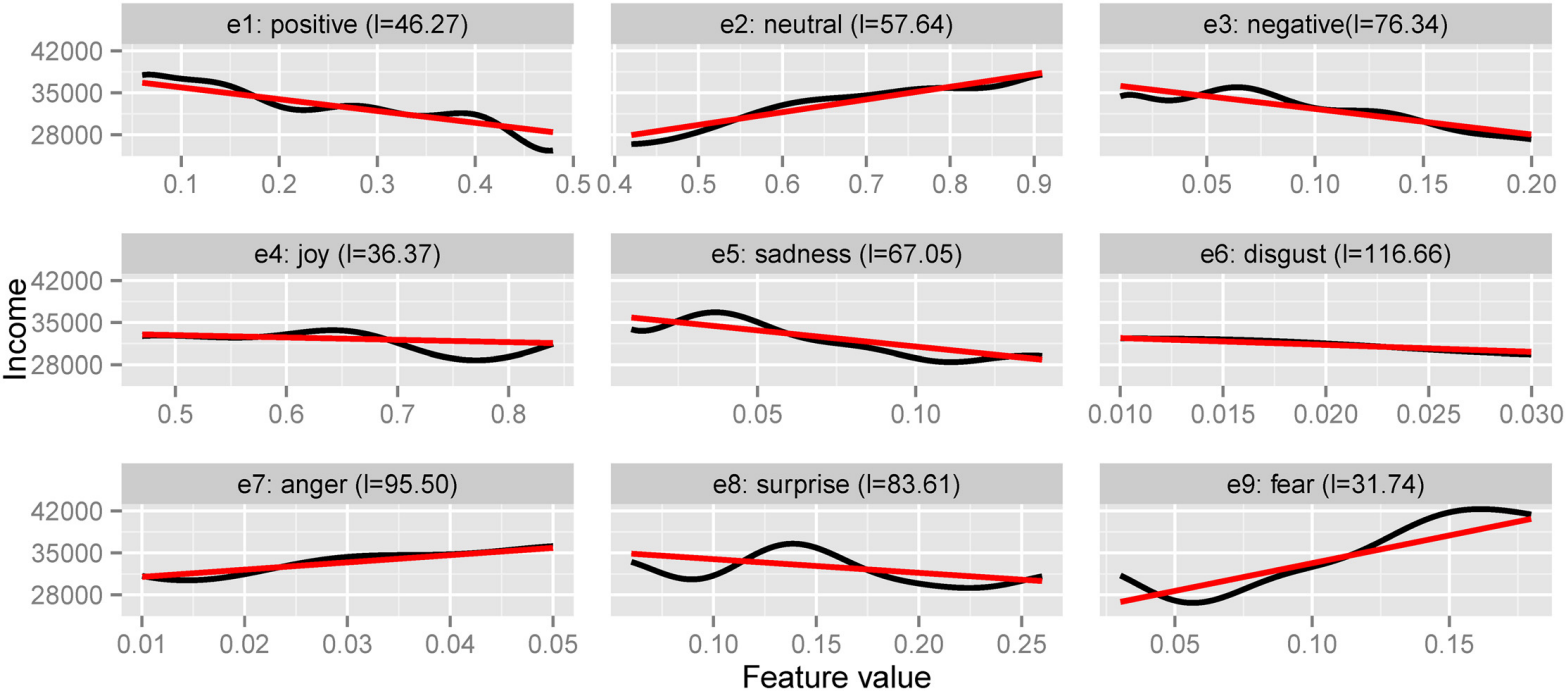
Linear and non-linear (GP) fit for emotions and sentiments. Variation of income as a function of user emotion and sentiment scores. Linear fit in red, non-linear Gaussian Process fit in black. Brackets show the GP lengthscales—the lower the value, the more important the feature is for prediction.

In Fig 4 we plot changes in emotions and sentiments of users with income. We find the following relationships:

- Neutral sentiment increases with income, while both positive and negative sentiment decrease. This uncovers that lower income users express more subjectivity online;
- Anger and fear emotions are more present in users with higher income while sadness, surprise and disgust emotions are more associated with lower income; the changes in joy are not significant.

Similarly to our analysis between income and psycho-demographics, we test whether these emotional changes are statistically significant using a Mann-Whitney test on the 1,000 user profiles with highest and lowest income. We found that all differences in means between these two groups of users are statistically significant (*p* < .001), except for joy. The differences in mean values between these two groups of users as the income increases are: positive 5%↓, negative 4%↓, neutral 9%↑, sadness 2%↓, disgust 0.25%↓, anger 0.27%↑, surprise 1.5%↓ and fear 3.3%↑.

### Shallow Textual Features

**Fig 5:**
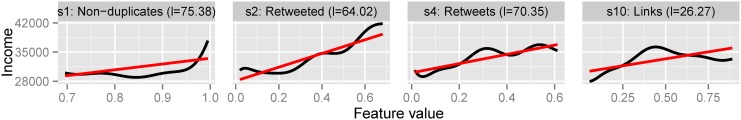
Linear and non-linear (GP) fit for shallow textual features. Variation of income as a function of user shallow textual features. Linear fit in red, non-linear Gaussian Process fit in black. Brackets show the GP lengthscales—the lower the value, the more important the feature is for prediction.

Fig 5 shows the shallow textual features against user income for the top five most predictive features as determined by the GP lengthscales. The rest of the features have very high lengthscales (higher than all profile features for example) and do not exhibit any observable patterns.

The following relationships are identified:

- Lower income users use more URLs (*s*_10_) in their posts, showing that these users link to external content such as news, pictures or videos;
- Higher income users get retweeted more (*s*_2_) and also perform many retweets (*s*_4_) themselves. This points out that high income users use Twitter more for content dissemination. This is affected also by the larger number of followers higher income users have, which raises the likelihood of a tweet to be retweeted;
- Although the majority of users do not post duplicate content (*s*_1_), those who do have lower income.

### Topics

The word clusters allow to gain insights into text use and its relation to income. We assume there is a variation in language between the entire spectrum of user incomes [16, 55]. We also note that the GP lengthscales for the top 10 topics are lower (i.e. more predictive) than all except three features from the Profile, Emo and Shallow categories combined. This further confirms the good predictive power of the word clusters. Table 4 shows ten of the most informative topics represented by top 15 words, sorted by their ARD lengthscale (*l*).

**Table 4:** Topics, represented by top 15 words, sorted by their ARD lengthscale. Most predictive topics for income. Topic labels are manually added. Lower lengthscales (*l*) denote more predictive topics.

**Rank**	**Topic #**	**Label**	**Topic**	*l*
1	139	Politics	republican democratic gop congressional judiciary hearings abolishing oppose legislation governors congress constitutional lobbyists democrat republicans	3.10
2	163	NGOs	advocacy organization organizations advocates disadvantaged communities organisations participation outreach associations non-profit nonprofit orgs educators initiative	3.44
3	196	Web analytics / Surveys	#measure analytics #mrx #crowdsourcing crowdsourcing #socialmedia #analytics whitepaper #li metrics #roi startup #social #smm segmentation	3.68
5	124	Corporate 1	consortium institutional firm’s acquisition enterprises subsidiary corp telecommunications infrastructure partnership compan aims telecom strategic mining	6.48
6	91	Corporate 2	considerations provides comprehensive cost-effective enhance advantages selecting utilizing resource essential additionally specialized benefits provide enhancing	7.44
7	107	Justice	allegations prosecution indictment alleged convicted allegation alleges accused charges extortion defendant investigated prosecutor sentencing unlawful	7.84
8	92	Link words	otherwise unless wouldn’t whatever either maybe pretend anyone’s assume eventually assuming or bother couldn’t however	8.39
9	173	Beauty	hair comb bleached combed slicked hairs eyebrows ponytail trimmed curlers dye dyed curls waxed bangs	9.75
10	40	Sport shows	first-ever roundup sport’s round-up rundown poised previewing spotlight thursday’s com’s long-running joins concludes prepares observer	10.57
11	99	Swearing	messed f’d picking effed cracking f*cked hooking tearing catching lighten picked cracks ganging warmed fudged	11.09

**Fig 6:**
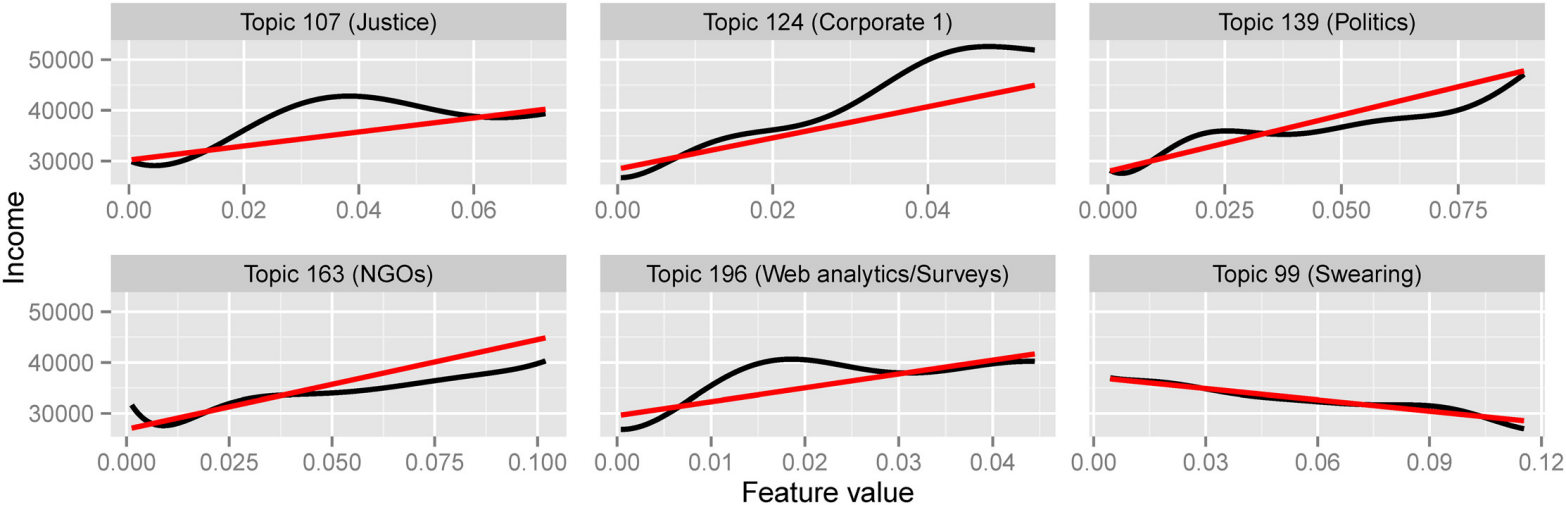
Linear and non-linear (GP) fit for topics. Variation of income as a function of user topic usage. Linear fit in red, non-linear Gaussian Process fit in black.

In Fig 6 we plot the linear and Gaussian Process fit for a selection of important topics.

These cover a broad range of thematic subjects:

- First, users talk more about ‘Politics’ (Topic 139) as the income gets higher in a near linear relationship. Since income is usually strongly associated with the level of education, we observe that higher educated users talk more about politics than less educated ones. Another reason is that richer people are closer to political elites and therefore are more concerned about politics;
- A similar near linear relationship is observed between tweeting about ‘NGOs’ (Topic 163) and ‘Corporate’ (Topic 124), with higher income users using these topics more. NGOs are usually supported by donations which are usually made by people with higher incomes. Therefore, even if NGOs activities are of general interest, they concern and are more discussed by users that participate actively in these organisations. Furthermore, tweeting about ‘Corporate’ is expected to be more prevalent amongst corporate members and investors who usually have higher than average income;
- On the other hand, an opposite linear relationship is present for ‘Swearing’ (Topic 99). Swear words are used more by people with lower incomes which suggest that they use more informal language. The same behaviour holds in general with topics that contain more personal language or words with alternative spellings (topics not shown here). This suggests that in general users of lower income use social media more for personal communication, while the ones with higher incomes use it for more ‘professional’ issues;
- Some topics reveal non-linear relationships with income which shows the effectiveness of modelling this task using GPs. For example, users with higher incomes tweet more about ‘Web analytics / Surveys’ (Topic 196) and ‘Justice’ (Topic 107) with a plateau above a certain feature value. This shows that using these topics helps discriminate higher from lower incomes, but the larger the volume of tweets on these topics does not imply even higher income.

### Limitations

In our study, we showed that it is feasible to gather a large dataset with little cost. However, we acknowledge possible limitations. Deriving income statistics from job labels is not perfect, although variance withing the 3-digit SOC groups is small. We were constrained by using the UK income levels for our outcome based on their mapping to the SOC classification. Our users, however, are not geolocated, as this would decrease our dataset size drastically—only 1–2% tweets or users are geolocated and represent a biased sample [56], profile field geolocation is not perfect or very frequent [57] and automatic geolocation prediction methods [58] would introduce biases in our analysis. Self-identification of the occupation is potentially another source of bias, albeit we were able to find users representative to most job types. Notably, the self-identification approach is being used widely throughout the literature [58–60].

For the psycho-demographic analysis we relied on predictions from automated text-based algorithms. The majority of these were trained on perceived annotated ground truth labels, leading the classifier to estimate the perception of those traits from text. A future study that has access to better data could study the interplay between income and real user psycho-demographic traits as measured using questionnaires.

## Conclusions

We presented the first large-scale study aiming to predict the income of social media users from their generated content and online behaviour. Framing this task as a regression problem, we demonstrated high predictive power using a combination of publicly available features, such as language and profile data, with automatically inferred characteristics from text, such as perceived psycho-demographics, emotions and sentiment.

The interpretability of the applied Gaussian Process model allowed us to perform an extensive qualitative and quantitative analysis of the input features. We found that the proportion of tweets using vocabulary related to fear or joy, the ratios of tweets with links and retweets as well as topics discovered in the textual content have high predictive power. We also discovered that users perceived to be female, younger, African American, with lower education level, or anxious are associated with lower rates of income. On the other hand, users with higher income post less emotional (positive and negative) but more neutral content, exhibiting more anger and fear, but less surprise, sadness and disgust. Finally, through an analysis on user language, we were able to highlight latent topics that discriminate users with high and low income, such as politics, specific technology topics or swear words.

Acknowledging possible limitations of this study, we consider our study as a necessary first step in analysing income through social media using datasets orders of magnitude larger and with more complex features than previous research efforts. We believe that the presented methods can find several applications in a multitude of domains, ranging from health to politics or marketing.
